# Prevalence of soil transmitted nematodes on Nukufetau, a remote Pacific island in Tuvalu

**DOI:** 10.1186/1471-2334-6-110

**Published:** 2006-07-12

**Authors:** Rick Speare, Falatea Fab Latasi, Tekaai Nelesone, Sonia Harmen, Wayne Melrose, David Durrheim, Jorg Heukelbach

**Affiliations:** 1 Anton Breinl Centre for Public Health and Tropical Medicine, WHO Collaborating Centre for Lymphatic Filariasis, James Cook University, Townsville 4811, Queensland, Australia; 2 Ministry of Health, Funafuti, Tuvalu; 3 Health Protection, Hunter New England Population Health, Locked Bag 10, Wallsend 2287, New South Wales, Australia; 4 Department of Community Health, School of Medicine, Federal University of Ceará, Fortaleza CE 60430-140, Brazil

## Abstract

**Background:**

The population of Nukufetau, a remote coral atoll island in Tuvalu in the Western Pacific, received annual mass drug administration (MDA) of diethylcarbamazine and albendazole under the Pacific Elimination of Lymphatic Filariasis program in 2001, 2002 and 2003, with the last MDA occurring six months before a cross-sectional survey of the whole population for soil transmitted helminths (STH).

**Methods:**

A cross-sectional survey in May 2004 recruited 206 residents (35.2% of the population) who provided a single faecal sample that was preserved, concentrated and examined microscopically.

**Results:**

Overall prevalence of STH was 69.9%; only hookworm and *Trichuris trichiura *were diagnosed. *Trichuris *was present in 68.4% with intensity of infection being light in 56.3%, medium in 11.7% and heavy in 0.5%. Hookworm occurred in 11.7% with intensity of infection 11.2% being light and medium in 0.5%. Twenty individuals (9.7%) had dual infections. The prevalence of *Trichuris *was constant across all ages while the prevalence of hookworm was significantly lower in residents below 30 years of age. In the age group 5–12 years comparison of results with a 2001 survey [1] suggested that the prevalence of STH has declined minimally, due to sustained high prevalence of *Trichuris*, while hookworm has declined dramatically from 34.4% to 1.6%.

**Conclusion:**

The results of this survey suggest that although the MDA appears to have reduced hookworm prevalence in residents below 30 years of age, there has been minimal effect on *Trichuris *prevalence. An integrated program to control STH is required.

## Background

Infections with soil transmitted helminths (STH) are common worldwide, with prevalence and parasite burden being particularly high in developing countries among school-aged children [2], although in many communities the prevalence of hookworm is higher in adults than in children [3]. In children, STH are associated with anaemia, stunting, underweight and poor school performance [4,5].

**Figure 1:**
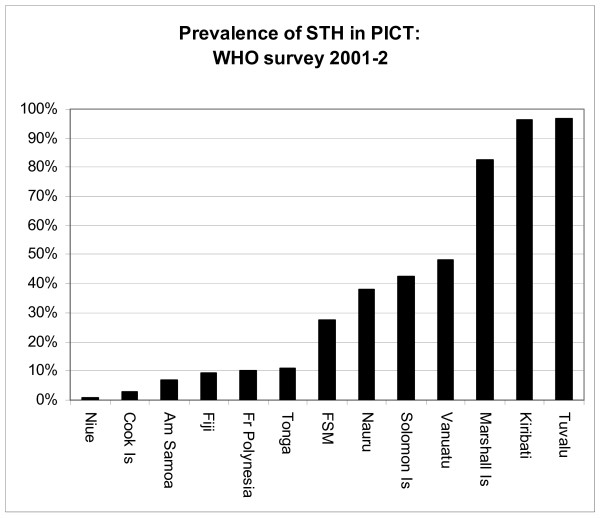
Prevalences of soil transmitted nematodes in children aged 5–12 years in PICT in 2001–2 (data reanalysed from Hughes et al 2004 [1] table 4).

A survey of STH in 13 Pacific island countries and territories (PICT) in 2001–2 found a very narrow spectrum of nematode parasites in children with a wide range of prevalences [1]. In this survey, prevalences in children aged 5 to 12 years ranged between 1% and 97% with prevalences lower then 5% in Niue and the Cook Islands, but very high prevalences (>80%) on the Marshall Islands, Kiribati, and Tuvalu (Fig 1). The reasons for this variation are not known.

In Tuvalu, samples from 118 children were analyzed, with 32 of these from the island of Nukufetau. Tuvalu had the highest prevalence (97%) of the 13 PICT, and only hookworms and whipworms (*Trichuris trichiura*) were detected [1]. There was not a single case of *Ascaris *infection. The species of hookworm was not identified. Two schools were surveyed, one on the main island of Funafuti with a resident population of 4,452, and another on the remote island of Nukufetau, with a resident population of 585.

Tuvalu had no programs or strategies to control STH before 2001, but in 2001, after the faecal survey, annual mass drug administration (MDA) of diethylcarbamazine (DEC) and albendazole was commenced for lymphatic filariasis elimination [6]. MDA was administered to all residents aged two years and older except for pregnant women and people considered too ill due to other diseases. The coverage (number of residents who received and consumed the drugs/total population) for Tuvalu was 81% in 2001, 47% in 2002 and 83% in 2003 [6].

This paper reports the results of a survey for STH amongst all age groups of Nukufetau residents in 2004, three years after the previous school-based survey [1] and following three rounds of MDA using albendazole and DEC. The survey was an opportunistic one, not linked to the MDA, but initiated to establish the need for a STH control program. The latest MDA had occurred six months prior to this survey.

## Methods

A cross-sectional survey was conducted on the island of Nukufetau, Tuvalu. Nukufetau is located at S8°15' E178°22', approximately 110 km north west of Funafuti, the capital island of Tuvalu. At the time of the last census in 2002, the island had 585 residents. All islands in Tuvalu are coral atolls.

In May 2004, all residents of Nukufetau were provided with containers for collection of faeces. To each specimen returned, SAF (sodium acetate, acetic acid and formaldehyde) solution was added, approximately equal to the volume of the faecal sample, and the faeces macerated to form a slurry. The fixed samples were transported to Princess Margaret Hospital Laboratory (PMHL) on Funafuti for examination. Details recorded for each sample were the resident's name, age and gender.

At the PMHL approximately 0.2 g of faeces was placed in a plastic conical 10 ml centrifuge tube with 8 ml of 10% formalin and 1 ml of petrol, and vigorously shaken by hand. The tubes were then centrifuged for 10 min at roughly 500 G. The ring of petrol and associated debris was loosened in each tube and all fluid in the tube tipped out. Volume was then reconstituted to 1 ml using tap water, mixed and 0.05 ml examined under two cover-slips (24 × 24 mm) placed side by side on a microscope slide at ×100 magnification using a compound light microscope. One slide was prepared from each specimen. Results were reported as number of eggs seen per slide and then calculated as eggs per gram (EPG) by multiplying by 200. The multiplication factor was determined by dilution of original sample at fixation (×2), 0.2 g in test tube (×5), examination of 0.05 ml of 1 ml (×20). Egg results were reported as light, medium and heavy using the criteria of WHO [7]: an egg count of 1–999, 1000–9999 and ≥9999 EPG was considered as light, moderate and heavy infection for *T. trichiura*, and 1–1999, 2000–3999 and ≥3999 EPG as light, moderate and heavy infection for hookworms.

Data was entered into an Excel file, checked for errors that may have occurred during data entry and analysed using SPSS for Windows (version 12.0.1) to generate descriptive statistics. Chi-square tests were used to test for differences in prevalence by gender and age category. Geometric mean intensity of EPG for *Trichuris *was calculated for all participants. The study was performed under James Cook University Ethics Committee Approval number H1423 and with the permission and full involvement of the Ministry of Health, Tuvalu.

## Results

Two hundred and six specimens were collected, 35.2% of the population of Nukufetau. Males provided 96 specimens (46.6%) and females 110 (53.4%). Mean age was 33 years with a median of 34 years and a range from seven months to 84 years. All age-groups were represented (Table 1) with 20–29 years under-represented at 11.8% and >49 years over-represented at 60.5%. STH were diagnosed in 69.9%. (Table 1).

**Table 1:** Prevalence of STH in Nukutefau residents by age-group, 2004. Population data are from 2002 census.

Age group (years)	Total population	Number examined (% pop of age group)	Hookworm positive (%)	*Trichuris *positive (%)	STH positive (%)	*Trichuris *Geometric Mean Intensity EPG*
0–9	171	62 (36.3)	1 (1.6)	43 (69.4)	43 (69.4)	54.2
10–19	95	26 (27.4)	1 (3.8)	19 (73.1)	19 (73.1)	67.6
20–29	51	6 (11.8)	0 (0.0)	5 (83.3)	5 (83.3)	40.9
30–39	70	15 (21.4)	3 (20.0)	10 (66.7)	10 (66.7)	35.3
40–49	79	25 (31.6)	5 (20.0)	16 (64.0)	17 (68.0)	17.6
50–59	52	37 (71.2)	9 (24.3)	27 (73.4)	27 (73.0)	56.0
60–69	47	23 (48.9)	3 (13.0)	13 (56.5)	14 (60.9)	20.3
≥70	20	12 (60.0)	2 (16.7)	8 (66.7)	9 (75.0)	35.5
Total	585	206 (35.2)	24 (11.7)	141 (68.4)	144 (69.9)	41.2
CI 0.95			9.5–13.9%	65.2–71.6%	66.7–73.1%	

*Geometric Mean Intensity EPG calculated for all subjects as per Mani et al (2004).

Only hookworm and *Trichuris *infections were diagnosed. The overall prevalence of hookworm was 11.7% with a prevalence of 2.1% in residents under 30 years of age and 19.6% amongst residents 30 years of age or older (Table 1). There was a significant difference in prevalence by age category (p = .014). The overall prevalence of *Trichuris *was 68.4% with no significant difference in prevalence by age group. Twenty (9.7%) residents had concomitant infections with hookworm and *Trichuris*, and only three were infected by hookworm alone. There was no significant difference in prevalence of *Trichuris *or hookworm infections by gender.

Amongst the 61 children aged 5–12 years, 46 (75.4%) had STH, all with *Trichuris *and one (1.6%) had a concomitant infection with hookworm. Fifty percent (12/24) of the children less than 5 years had STH including 25% of the 12 children 2 years of age and younger. Only one child under 5 years, a 4 year old, had hookworm infection. This child also had *Trichuris *as did the remaining 12 STH positive children. The youngest child with *Trichuris *was 1 year of age.

For individuals with *Trichuris *infection the mean (±std) and maximum EPG were 673 (±1510) and 15,200 respectively. The proportions with light, medium and heavy infections were 56.3%, 11.7% and 0.5% respectively. For hookworm the mean (±std) and maximum EPG were 242 (±407) and 2,040 respectively. The proportions with light and medium infections were 11.2% and 0.5% respectively. There were no heavy infections. For the 61 children 5–12 years of age surveyed, intensity of infection with *Trichuris *was light in 57.4%, medium in 16.4% and heavy in 1.6%.

## Discussion

To the best of our knowledge, this is the first published cross-sectional prevalence survey of STH amongst an entire island population in the Western Pacific Region. The prevalence of STH in this remote atoll island was high (69.9%), mainly due to infection with *T. trichiura*, and the spectrum of geohelminths was narrow. Trichuriasis was equally distributed across all ages while the prevalence of hookworm infection was low (2.1%) under 30 years of age, but 19.6% in residents aged 30 years or older. Since the species of hookworm in Tuvalu is unknown, the high prevalence warrants studies to determine the species and to evaluate the health impact of hookworm infection, particularly the possible occurrence of iron deficiency anaemia in reproductive age and older women. Interestingly, not a single case of *Ascaris *infection was detected, confirming Hughes et al's findings for Tuvalu [1].

The only available baseline data on STH for the population of Nukufetau prior to the MDA campaigns comes from the survey amongst children aged 5–12 years in 2001 [1]. Although the prevalence of STH in this age range has changed only slightly in Nukufetau since 2001, this is due to the high prevalence of *Trichuris*. In the survey in 2001 in the age group 5–12 years, prevalence of *Trichuris *was 84% (n = 32) while in our survey prevalence was 75% (n = 61), a decline in prevalence of 11%. However, hookworm has declined dramatically in this age-group from 34.4% to 1.6%, a 95% reduction in prevalence. Intensity of infection with hookworm has remained constant with all cases in the light category [1]. Intensity of *Trichuris *in this age group has not changed significantly, with light 59.4% and medium 25% in 2001 and light 57.4%, medium 16.4% and heavy 1.6% in this survey. However, direct comparison is difficult since the participants in neither survey were randomly selected [8]. Only one of the 24 children under 5 years, a 4 year old, had hookworm, a prevalence of 4.2% for children less than 5 years of age. Children less than 3 years at the time of this survey had not participated in any MDA round, while those aged 3, 4 and 5 years and older had possibly participated in one, two and three MDA rounds respectively. The transmission of hookworm may have decreased as a result of the annual MDA campaigns.

MDA with DEC and albendazole achieved a reduction in hookworm prevalence in Indonesia from 25.3% to 5.9% [3]. Although on Nukufetau the hookworm prevalence in adults was high, without a pre-MDA baseline amongst adults comments on trends cannot be made. The initial hookworm prevalence was 50% in Indonesian residents older than 50 years and demonstrated a dramatic reduction in this age group after two rounds of MDA [3]. A single dose of 400 mg albendazole achieved an average cure rate of 78% in 68 studies [9]. Hence, for Nukufetau after albendazole had been administered annually for three years, a lower prevalence would have been expected.

The coverage of MDA in Nukufetau, at least in the first and third round, was very good. However, compliance with the MDA may have affected the success of treatment, especially in the middle and older age groups, which showed higher prevalences of hookworm infection. Unfortunately, individual data on compliance was not available. However, at the mass treatment rounds, men were more likely to be unavailable for observed treatment owing to their being absent while fishing or travelling.

Rate of re-infection after treatment can be considerable [10], but the low prevalence of hookworm in children suggests that incidence is low and that re-infection is unlikely to account for the high adult prevalence. *Trichuris *eggs can survive for extended times in the environment as compared to hookworm eggs and larvae, which may have contributed to a higher prevalence of trichuriasis than hookworm infection after mass treatment. Additionally, hookworm may have developed resistance to albendazole. However, resistance of human hookworms to anthelmintics has not been definitely reported. Resistance to pyrantel was suspected in Australia in *Ancylostoma duodenale *with the strain susceptible to albendazole [11]. Reduced efficacy of mebendazole was reported in Mali [12] and after 5 years of intensive therapy in Zanzibar [13]. Although anthelmintic resistance is less likely to account for persistence of hookworm than non-compliance in MDA, standardised resistance testing should be conducted on this population of hookworms in Nukufetau [14,15].

This study highlights the persistence of *Trichuris *infection after three rounds of MDA using albendazole and DEC. Studies from Haiti, India and Sri Lanka reported in contrast to our data reduction of the prevalence of trichuriasis after mass treatment with albendazole and DEC [16-18]. DEC has no significant activity against hookworm and *Ascaris lumbricoides *[19] and is unlikely to be active against *T. trichiura*. Albendazole has limited activity against *Trichuris *with prevalences after the standard single dose of 400 mg declining between 23% to 48% [9,20,21] or showing no effect at all [10]. The prevalence of *Trichuris *was reduced between 7% and 48%, the latter from a cohort study, of *Trichuris *in Indonesia after two rounds of MDA using albendazole and DEC [3]. A cure rate of 30% with the same anthelminthic combination against *T. trichiura *was reported in Sri Lanka [22]. Ongoing transmission despite repeated MDA campaigns in Nukufetau is suggested by the high rate (66%) of *Trichuris *in children under 5 years of age. Another anthelminthic, such as mebendazole or ivermectin, which has greater efficacy against *Trichuris *than albendazole should be used in Tuvalu to eliminate *Trichuris*. Mebendazole has been reported to have a high efficacy against *Trichuris *infections [23]. The data on efficacy of single dose ivermectin for *T. trichiura *are inconsistent with cure rates ranging from 0% to 88% [24-28], and two doses of ivermectin showed cure rates between 84% and 100% [29,30]. Ivermectin with albendazole had a higher cure rate than albendazole alone (79% versus 30% respectively) [22].

The PICT share particular epidemiologic and ecological features that may have contributed to these different results. For example, the parasite fauna is different from other parts of the world, which is exemplified by the complete absence of ascariasis and the high prevalence of trichuriasis in our study population. Different strains with different susceptibilities to anthelmintic drugs may also prevail.

For Nukufetau chemotherapy should be combined with an integrated control strategy to reduce the contamination of the island with eggs [5]. Behaviours that lead to eggs gaining access to soil and surviving the two weeks required for development in the egg have not been investigated in PICT. Homes on Nukufetau have septic sewage systems, but the quality of systems is not high. Rain is the only available source of potable water and when rainwater tanks become depleted during the dry season, residents may be reluctant to use water for toilets and personal hygiene (TN, personal observation). An association between inadequate water supplies in schools and STH was found in 14 PICT [1]. Defecation on the beach or in the sea is not uncommon in Nukufetau (TN, personal observation). The survival of hookworm eggs and larvae and *T. trichiura *eggs in sea-water appear not to have been tested. Experimentally sodium chloride at 2% concentration and higher had a deleterious effect on development of hookworm eggs and larvae [31]; seawater contains approximately 3.5% sodium chloride. However, the embryo of *Trichuris*, protected by a thick shell, could potentially survive immersion in sea-water. This hypothesis warrants investigation as it has implications for management strategies for *Trichuris *in small island nations.

## Conclusion

We have shown that on a remote island in the Western Pacific the spectrum of STH present in the general population is quite narrow with only hookworm and *Trichuris *infections present and that the prevalence of STH remains high after three rounds of MDA with albendazole and DEC. It appears that the MDA for lymphatic filariasis elimination has resulted in a significant reduction in hookworm prevalence in residents under 30 years of age. However, we assume based on previous data in children aged 5–12 years [1] and the high prevalence overall that annual community DEC and albendazole administration has had a minimal effect on *T. trichiura *prevalence. Alternative anthelmintics plus measures to reduce environmental contamination with helminth eggs need to be considered as part of an integrated STH control program.

## Competing interests

The WHO Collaborating Centre for Lymphatic Filariasis received funding for filariasis research on Tuvalu from Glaxo Smith Kline, the manufacturers of albendazole.

## Authors' contributions

RS: Assisted in project design, conducted the survey, performed laboratory examination, did statistical analysis and wrote the initial draft of the manuscript. FFL: Assisted in project design, conducted the survey, performed laboratory examination and contributed to the manuscript; TN: Assisted in project design and contributed to the manuscript; SH: Contributed to the manuscript; WM: Assisted in project design and contributed to the manuscript; DD: Assisted in project design and contributed to the manuscript; JH: Contributed to the manuscript.

## Pre-publication history

The pre-publication history for this paper can be accessed here:


